# Quantitative proteomic analysis of bronchoalveolar lavage fluid in West Highland white terriers with canine idiopathic pulmonary fibrosis

**DOI:** 10.1186/s12917-022-03202-x

**Published:** 2022-03-30

**Authors:** Rosemary E Maher, Merita Määttä, Robert J Beynon, Henna P Laurila, Paul S McNamara, Minna M Rajamäki

**Affiliations:** 1 Centre for Proteome Research, Institute of Systems and Integrative Biology, University of Liverpool, Liverpool, United Kingdom; 2 Department of Equine and Small Animal Medicine, Faculty of Veterinary Medicine, University of Helsinki, Helsinki, Finland; 3 Department of Child Health (University of Liverpool), Institute in the Park, Alder Hey Children’s Hospital, Eaton Rd, L12 2AP Liverpool, United Kingdom

**Keywords:** Bronchoalveolar lavage fluid, Dog, Lung, Reflux aspiration, Canine, Reflux aspiration, Proteomics: West Highland white terrier, CIPF

## Abstract

**Background:**

Canine idiopathic pulmonary fibrosis (CIPF) is a chronic, progressive, interstitial fibrosing lung disease, manifesting as cough, exercise intolerance and ultimately, dyspnea and respiratory failure. It mainly affects West Highland white terriers (WHWTs), lacks curable treatment and has a poor prognosis. Aspiration of gastroesophageal refluxate may play a role in the development of CIPF. In the first part of this study, we completed label-free quantitative proteomic analysis of bronchoalveolar lavage fluid (BALF) from CIPF and healthy WHWTs. In the second part, we evaluated potential protein markers of reflux aspiration from canine gastric juice and vomitus and whether these were present in BALF from the two groups.

**Results:**

Across all BALF samples, 417 proteins were identified, and of these, 265 proteins were identified by two or more unique tryptic peptides. Using the 265 high confidence assignments, the quantitative proteome profiles were very similar in the two cohorts, but they could be readily resolved by principal component analysis on the basis of differential protein expression. Of the proteins that were differentially abundant in the two groups, several (including inflammatory and fibrotic markers) were elevated in CIPF, and a smaller, more diverse group of proteins were diminished in CIPF. No protein markers indicative of reflux aspiration were identified.

**Conclusions:**

Label-free proteomics allowed discrimination between CIPF and healthy WHWTs, consistent with fibrotic process but did not provide clear evidence for gastrointestinal aspiration. The measurement of proteins may provide a proteomics signature of CIPF that could be used to evaluate treatment options.

**Supplementary Information:**

The online version contains supplementary material available at 10.1186/s12917-022-03202-x.

## Background

Canine idiopathic pulmonary fibrosis (CIPF) is a chronic, progressive, interstitial fibrosing lung disease affecting mainly West Highland white terriers (WHWTs) characterized by unclear etiology, lack of curable treatment and poor prognosis [1–3]. Typical clinical signs include cough, exercise intolerance and, in later stages, dyspnea and respiratory failure [1, 2]. Similarities between CIPF and human idiopathic pulmonary fibrosis (IPF) and nonspecific interstitial pneumonia exist both in pathological and clinical findings [2, 4, 5]. Since WHWTs are susceptible to CIPF, it is likely that genetics, together with other putative risk factors such as gastroesophageal reflux and reflux aspiration, play a role in the development of CIPF [6].

Extra-esophageal reflux is thought to be common in IPF with silent and repetitive microaspiration of refluxate postulated to cause alveolar epithelial injury, dysregulated wound healing and fibrosis [7]. Detection of reflux aspiration is often based on evaluation of gastrointestinal contents in airway samples [8, 9]. In IPF, pepsin is considered an important potential marker of reflux aspiration [10, 11]. Previously, we have shown that bile acids, also considered a marker of reflux aspiration [8], were measurable in bronchoalveolar lavage fluid (BALF) in most CIPF and healthy WHWTs but not in healthy beagles [6]. Supporting a possible role of reflux aspiration in disease pathogenesis, Fastrès et al. [12] found bacteria originating from ingested food or water (Brochothrix, Curvibacter, Pseudarcicella and Flavobacteriaceae genus) to be significantly more abundant in lung microbiota of CIPF and healthy WHWTs than in healthy dogs of other breeds.

To gain a better understanding of the etiology and pathogenesis of CIPF in WHWTs, we have completed a proteomics analysis of BALF from healthy and CIPF WHWT subjects. Proteomics permits a broad survey of global protein expression and can provide relative quantitative data that permit a comparison between the two subject groups. In brief, BALF samples were digested to convert proteins into peptides that were then resolved by high resolution reverse-phase chromatography and analysed by tandem mass spectrometry, to gain the identity and a quantification measure of each protein in the mixture. From this we were able to add a functional perspective on proteins enhanced in CIPF BALF. Furthermore, using samples of canine gastric juice and vomit, we explored whether proteins from the upper gastrointestinal tract were present in BALF from CIPF and healthy WHWTs and whether they might have potential as biomarkers of reflux aspiration.

## Results

### Proteomic profiling of BALF in WHWTs

**Fig. 1:**
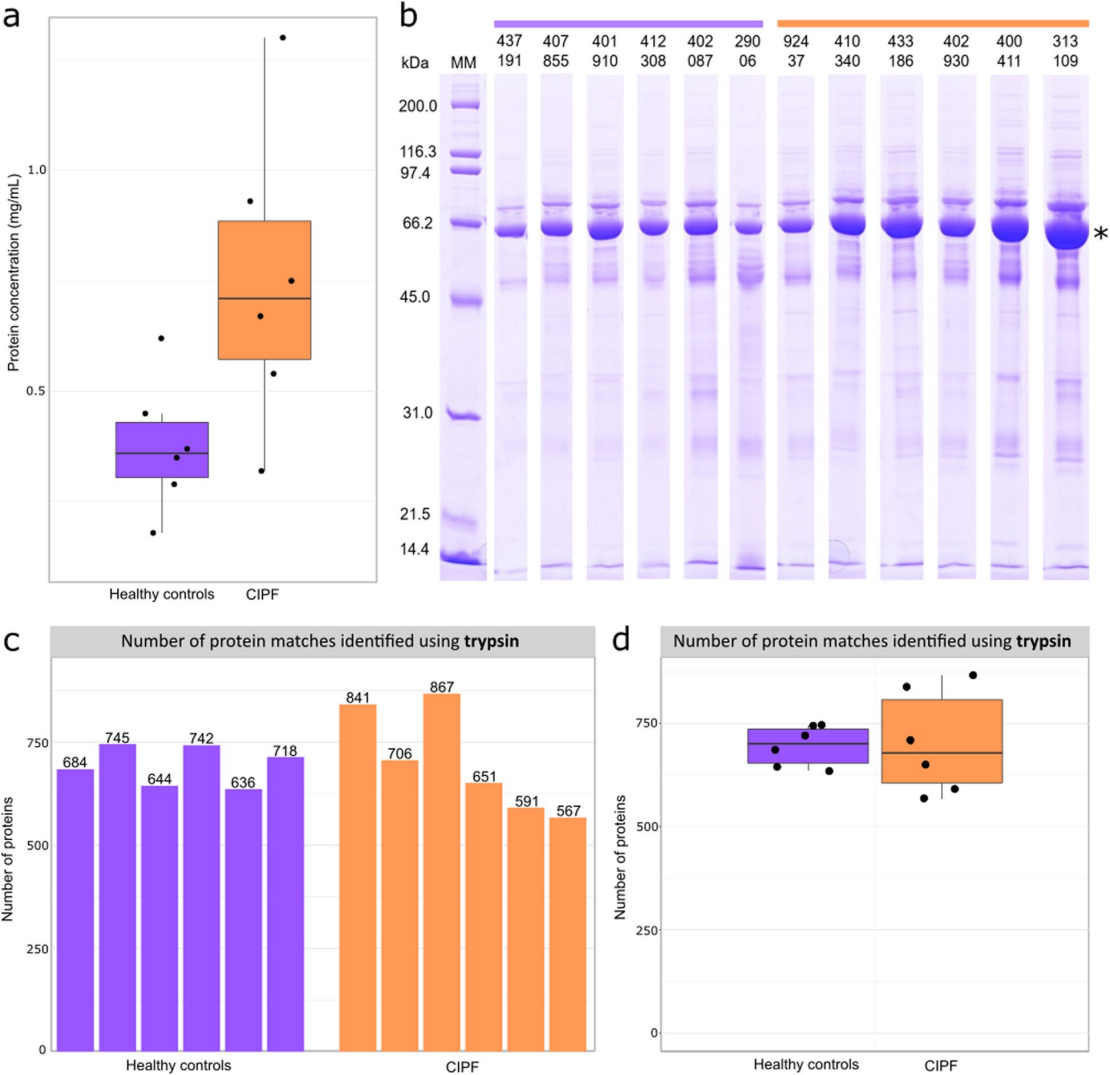
Protein identification in bronchoalveolar lavage fluid (BALF) of West Highland white terriers (WHWTs). For each sample, protein identification was performed on BALF from healthy control (*n* = 6) and CIPF (*n* = 6) WHWTs and is based on tryptic peptides. **a** Box plots compare the total protein concentration between healthy (purple) and CIPF (orange) WHWT BALF. Top and bottom of the box represent the 75% (Q3) and 25% (Q1) percentile, the line inside the box locates the median and whiskers define 1.5 times the interquartile range. **b** A 12% SDS-PAGE entire gel stained with Coomassie Brilliant Blue dye highlights protein bands in the control (purple) and CIPF (orange) WHWT BALF (10µL of each BALF sample). The band marked with an asterisk was subjected to in-gel digestion, confirming its identity as albumin. **c** Number of positive protein matches identified per sample based on tryptic peptides (summarised in **d**). Searches were completed through Proteome Discoverer/MASCOT at 1% FDR, based on one of more peptides for identification

Bronchoalveolar lavage samples from CIPF (*n* = 6) and healthy WHWTs (*n* = 6) were collected and used for comparative proteomics. The total protein concentration of BALF was increased in WHWTs with CIPF (mean ± SD, 0.75 ± 0.14 mg/mL) compared to healthy WHWTs (mean ± SD, 0.38 ± 0.06 mg/mL, *P* = 0.042, Fig. 1a). On SDS-PAGE, the protein profile of BALF was dominated by a high abundance of a protein that migrated at approximately 66 kDa (Fig. 1b), established as canine serum albumin by in gel digestion (results not shown).

**Fig. 2:**
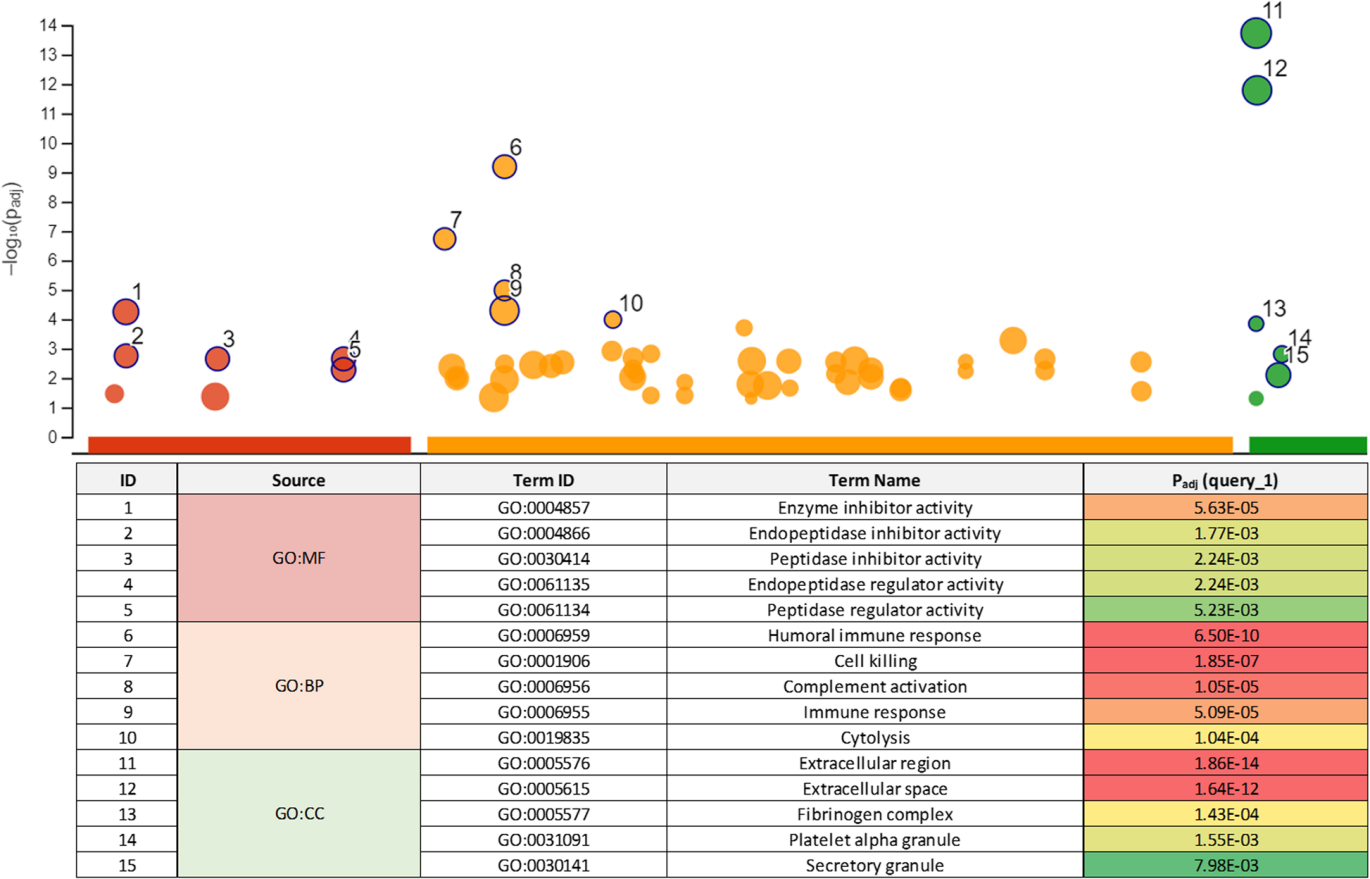
Functional analysis of the core bronchoalveolar lavage fluid (BALF) proteome in West Highland white terriers (WHWTs). The BALF ‘core’ proteome (265 proteins, present in BALF of both healthy WHWTs and WHWTs with canine idiopathic pulmonary fibrosis) was analysed with g:Profiler using default parameters. For the three GO categories (molecular function, GO:MF; biological process, GO:BP; cellular compartment, GO:CC) the top five over-represented terms are highlighted

Each BALF sample was digested with trypsin and subjected to proteomics analysis. Similar numbers of total proteins (approximately 700, range 567–857) were identified in all samples (Fig. 1c and d). When we applied the criterion that a protein should be identified in every sample, 417 proteins were observed in BALF across all twelve WHWT samples, based on one or more peptides for identification. Overall, there was little difference between the total number of proteins identified. To increase the confidence in protein identification, the 417 proteins were further filtered to require at least two unique peptides. By applying this filter, the number of proteins in the tryptic digests reduced to 265 high confidence assignments across all samples (we refer to this as the ‘core BALF proteome’). Functional enrichment analysis of this BALF core proteome highlighted the higher representation of secretory or exosomal proteins (Fig. 2), particularly in protease:antiprotease balance, complement and the humoral immune response.

**Fig. 3:**
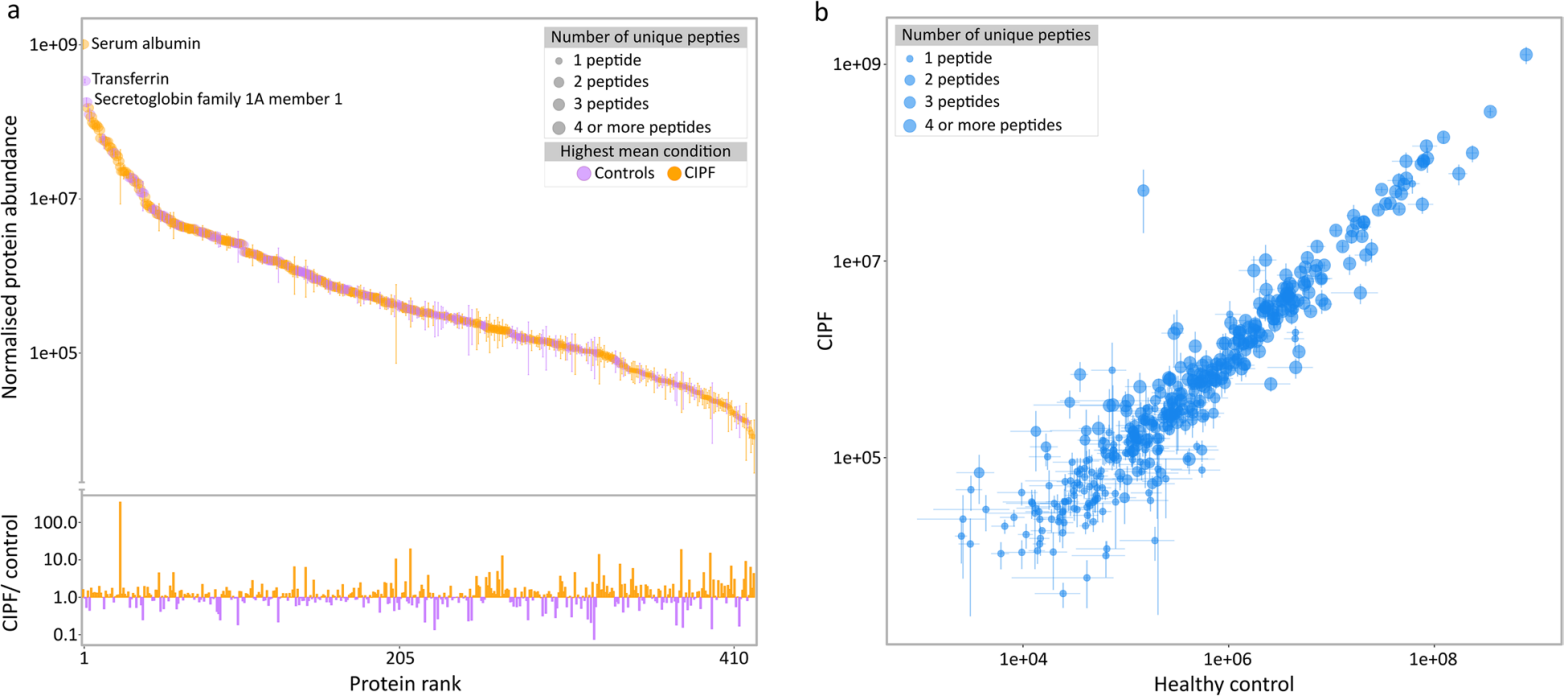
Quantitative profile of bronchoalveolar lavage fluid (BALF) in West Highland white terriers (WHWTs) proteins. Proteins from BALF of six control and six WHWTs with canine idiopathic pulmonary fibrosis were quantified by label-free methodology. All proteins were ranked and sorted by abundance (colour-coded, logarithmically scaled, panel a, top) and by differential expression (colour-coded, logarithmically scaled, (**a)**, bottom). When label-free abundance values for each protein were averaged and compared (**b**), there was a strong correlation between the two groups, with variance and poorer correlation being more prominent at the lower end of the scale. The error bars are the standard error of the mean for values in each group. Inset is the distribution of the number of peptides unique to each protein

Label-free quantification, based on the summed intensities of unique peptides derived from each protein, allowed the generation of a quantitative profile of the BALF proteins. As anticipated from the SDS-PAGE analysis (Fig. 1b), protein abundances spanned more than six orders of magnitude (Fig. 3a), although the 20 most abundant proteins explained 80% of the total label-free abundance. By far the most abundant protein was serum albumin. The next five most abundant proteins (Supplementary Table 1) were transferrin, uteroglobulin, also known as secretoglobin family 1 A member 1 (SCGB1A1), alpha 2-HS glycoprotein (fetuin A, AHSG), chloride channel accessory protein 1 (CLCA1) and haptoglobin.

The confidence in protein identification varied from protein to protein. Of the 417 proteins common to CIPF and healthy WHWTs BALF, 152 (36%) were based on a single unique peptide for identification (Fig. 3b). In the lower abundance region, protein identifications were achieved with fewer unique peptides, and the scatter in the data was more pronounced. The overall quantitative proteome profiles of the two conditions (control and CIPF) were very similar, as evidenced by the overlap between the log-transformed abundance waterfall plots (Fig. 3a) and the strong correlation between most protein abundances in the two conditions (Fig. 3a and b). This analysis thus satisfied the condition of an overall proteome similarity, creating a reference comparison from which differentially abundant proteins could be extracted.

### Comparison of the BALF proteome from control and CIPF samples

**Fig. 4:**
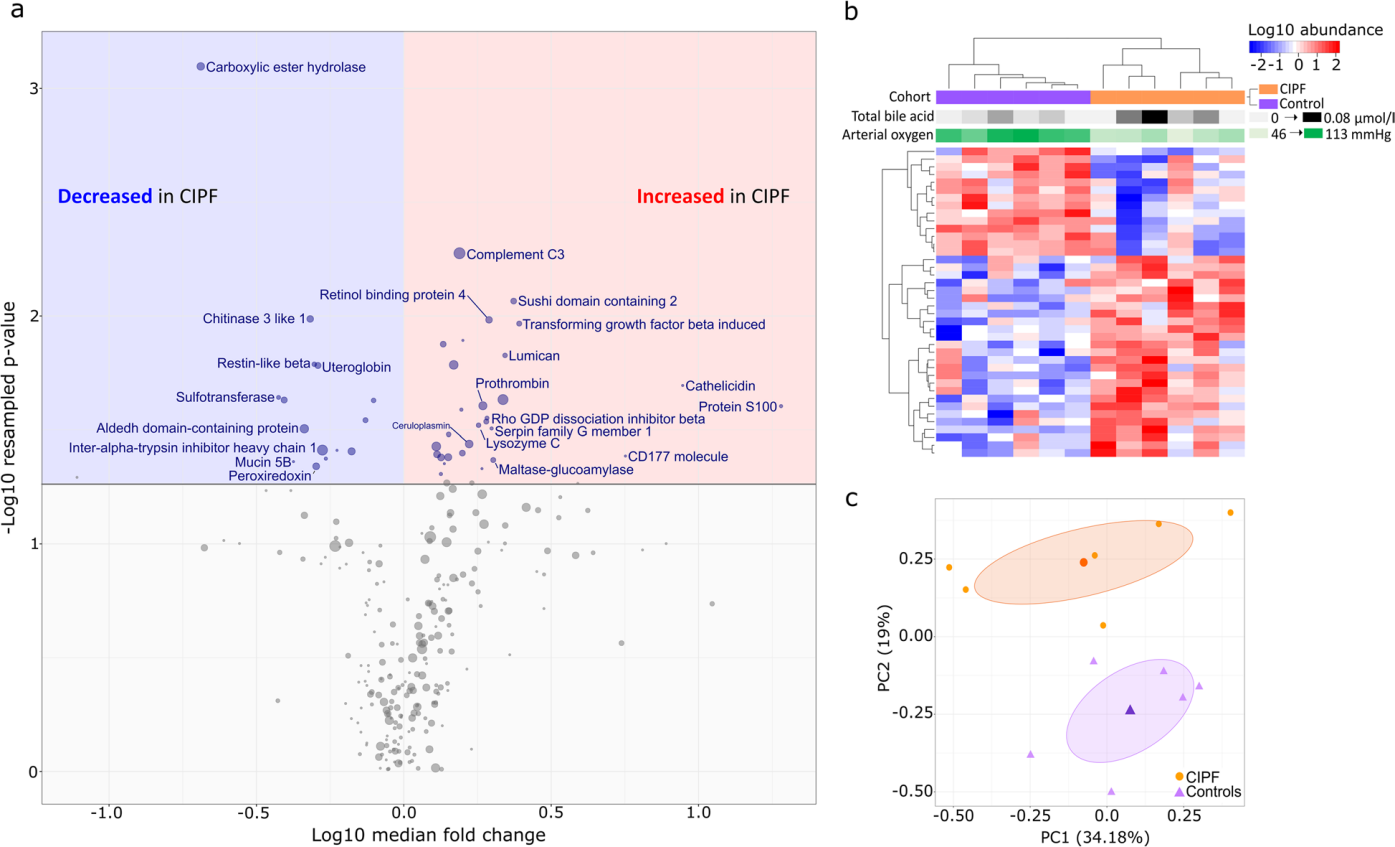
Quantitative comparison of the bronchoalveolar lavage fluid (BALF) proteomes in West Highland white terriers (WHWTs) with canine idiopathic pulmonary fibrosis (CIPF) and healthy WHWTs. Proteome differences between control and CIPF BALF were visualised using a volcano plot analysis of proteins identified with >1 unique peptide from trypsin analysis. The number of unique tryptic peptides identified is represented by the size of the symbol. Proteins above the grey line have a q-value < 0.05 (**a)**. Samples were clustered and displayed in a heat map of the scaled abundance for the top 40 differentially expressed proteins (based on fold difference), dog clinical condition, arterial oxygen and the concentration of total bile acids in BALF (**b)**. Details of the proteins are provided in Supplementary Figure 2. Data from arterial oxygen and total bile acids are from previous studies [2, 6]. The entire data set (265 proteins) was subjected to principal component analysis using all proteins identified with more than one unique peptide (**c)**

The BALF proteome was compared between CIPF and healthy WHWTs. For this analysis, the normalised abundance data from the core BALF proteome (265 proteins) were used. A volcano plot of the protein-level data revealed about 100 proteins that were significantly changed in CIPF BALF (resampled *P* < 0.05) (either up or down) relative to healthy WHWTs (Fig. 4a), although for many of these, the fold differences were small. When the top 40 most differentially expressed proteins, selected on the basis of the statistical confidence in the differential expression, were used to generate a heatmap, a clear segregation by hierarchical clustering between the two cohorts was evident, with proteins being either elevated or diminished in CIPF (Fig. 4b). Principal component analysis (PCA) of the normalized, z-scaled proteome data resolved CIPF and healthy WHWTs in the first two principal components with no overlap, explaining 34% and 19% of the variance respectively (total 53%, Fig. 4c).) There were clear proteome differences between BALF samples from healthy WHWTs and WHWTs with CIPF, although the CIPF group showed higher intragroup variability. The most obvious separation between healthy and CIPF groups was in PC2. The top 20 proteins that contributed to PC2, ranked according to the magnitude of the contribution are plotted in Supplementary Fig. 1.

**Fig. 5:**
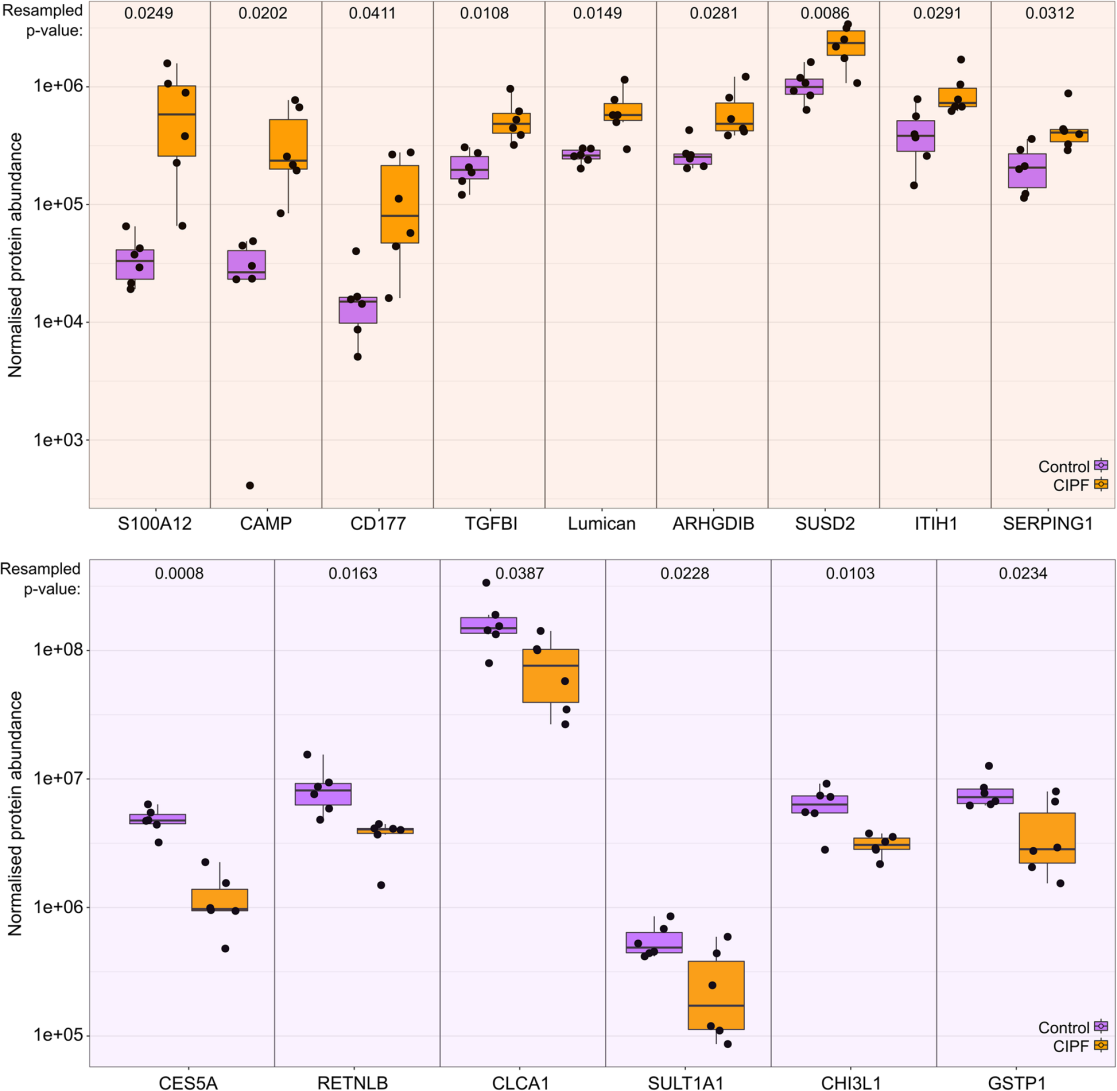
Expression of the proteins with the highest differential abundance in bronchoalveolar lavage fluid (BALF) in West Highland white terriers (WHWTs) with canine idiopathic pulmonary fibrosis (CIPF). The top fifteen proteins with a resampled P value < 0.05 were ordered by magnitude of fold change and were groups according to direction of change. Top: nine proteins that were elevated in BALF in CIPF and bottom: six proteins that were decreased in CIPF WHWTs. Label-free abundances for individual samples are plotted, together with a box and whiskers plot (median, box: 50 percentile limits, whiskers, 25th and 75th percentiles). The proteins were: S100A12, CAMP, cathelicidin; CD177; TGFB1, transforming growth factor beta induced ig-h3; lumican; ARHGDIB, Rho GDP dissociation inhibitor beta; SUSD2, sushi domain containing 2; ITIH1, inter-alpha-trypsin inhibitor heavy chain 1, SERPING1, Serpin family G member 1; CES5A, carboxylic ester hydrolase; RETNLB, putatively resistin-like beta; CLCA1, chloride channel accessory 1; SULT1A1, sulfotransferase; CHI3L1, chitinase 3 like 1 and GSTP1, glutathione S-transferase P1

The proteins with the largest magnitudes of change, either elevated or decreased in CIPF WHWTs, are presented in Fig. 5. The proteins that were most highly increased in CIPF WHWTs compared to healthy WHWTs included S100A12 (19-fold, Supplementary Table 1), cathelicidin (8.8-fold), Ly-6 like CD177 (5.6-fold), transforming growth factor beta 1 induced protein (2.4-fold), serpin G1 (2-fold) and inter-alpha trypsin inhibitor (1.9-fold). Additionally, all three fibrinogen chains were elevated between 1.8 and 3.8-fold in CIPF WHWTs. The proteins that were most significantly decreased in CIPF compared to healthy WHWTs include carboxylic acid ester hydrolase (5-fold), non-enzymatic chitinase 3 like 1 (2-fold) and secretoglobulin (2-fold).

### Protein evidence for reflux aspiration in WHWTs with CIPF

In the second part of this study, we used proteomics to discover potential proteins that could be indicative of reflux. First, a sample of gastric juice and one induced vomit sample from healthy dogs of other breeds were analysed to create a list of putative stomach-derived proteins. From gastric juice, 68 proteins were identified after trypsin digestion. Similarly, for the induced vomit sample, trypsin digestion revealed 63 proteins. Collectively, 126 proteins were identified in both gastric juice and induced vomit (Supplementary Table 2). Proteins of interest that might be used as potential markers of reflux aspiration were selected based on a high level of expression in the stomach. These included the trefoil factor family (TFF), gastrokines (GKNs), IgGFc-binding protein, pepsin A and gastric triacylglycerol lipase. Of note, these same proteins were identified in canine gastric juice in a separate study [13].

Trefoil factor 1 (TFF1) was detected with three unique peptides in digests of gastric juice and with two unique peptides in vomitus. Additionally, it was detected in trypsin digests of BALF by a single peptide and a cognate miscleavage product containing the same peptide. No significant difference was detected in TTF1 levels between CIPF and healthy WHWTs (*P* = 0.08). Trefoil factor 2 (TFF2) was detected with three unique peptides in gastric juice and with one unique peptide in vomitus but was not identified in BALF. Trefoil factor 3 (TFF3) was not identified in gastric juice or vomitus but was identified in tryptic digests of BALF. This protein was reduced in CIPF (*P* = 0.03). Gastrokine 2 (GKN2) was identified in gastric juice by three unique peptides. However, GKNs could not be identified in BALF from control or CIPF WHWTs. IgGFc-binding protein was identified in gastric juice with 11 unique peptides and vomitus (22 unique peptides). There was a broad distribution of values, but with no difference between the abundance of this protein in the two groups (*P* = 0.37). Pepsin A was detected in gastric juice by two peptides, and in induced vomit by a single peptide using trypsin digestion. We were not able to detect pepsin A in BALF samples of CIPF or healthy WHWTs. To explore the possibility of pepsin being present in BALF but not being identified because of the paucity of peptides, we generated extracted ion chromatograms for the candidate peptides but were unable to detect peptides from pepsin A in BALF of WHWTs (data not shown). Finally, gastric triacylglycerol lipase was identified in gastric juice with three unique peptides, but this protein was not detected in any BALF sample.

## Discussion

In the first part of this study, we evaluated BALF proteomic profiles in CIPF and healthy WHWTs. As an accessible biofluid for proteomics, BALF can yield several hundred protein identifications, although it has a relatively simple SDS-PAGE profile reflecting a strong bias to serum albumin. Functional enrichment analysis of BALF core proteome in WHWTs highlighted the higher representation of core BALF proteins as secretory or exosomal proteins, which is consistent with the fluid being rich in purposefully secreted proteins rather than because of cellular damage. In particular, the strongest scoring molecular function categories were associated with endopeptidase inhibition, consistent with the demand for regulation of ectopic proteolysis and modulation of coagulation/fibrinolysis, in addition to complement activation. As anticipated, the most abundant protein was serum albumin. The next five most abundant proteins were transferrin, AHSG, SCGB1A1, CLCA1 and haptoglobin. These high abundant proteins are a mixture of plasma proteins, presumably from plasma exudate in the respiratory/digestive system, and proteins that are elevated and secreted by lung tissue [14].

Additionally, we compared the proteome of BALF in CIPF and healthy WHWTs to find potential protein biomarkers for CIPF. A previous study explored BALF proteomic profile in CIPF WHWTs, dogs with chronic bronchitis (CB) and healthy dogs with 2-D differential gel electrophoresis [15]. In this study, six proteins (complement C3, α-1-antitrypsin, apolipoprotein A-1, haptoglobin, β-actin and transketolase) were significantly upregulated in both CIPF and CB dogs compared with healthy dogs. However, no specific CIPF biomarkers were identified. In our study, we observed proteomic differences between BALF of CIPF and healthy WHWTs. The CIPF group showed higher intragroup variability, probably reflecting the variability in disease presentation by individual dogs. Expression of several proteins was increased in CIPF compared to healthy WHWTs. One of these, the neutrophil-derived protein S100A12, is a major modulator of the inflammatory response [16]. Elevated expression of cathelicidin and Lys-6 like CD177 in CIPF WHWTs is consistent with an activation of inflammatory response and of neutrophil function [17, 18]. Elevated TGF-β1 induced protein is consistent with an effect on cell proliferation and the onset of fibrotic changes [19, 20]. Increased TGF-β1 signaling has also been detected previously in CIPF WHWTs compared with healthy controls and TGF-β is considered a key mediator in CIPF of WHWTs [21, 22]. Elevation of serpin G2 and inter-alpha trypsin inhibitor are consistent with a proteinase:antiproteinase reaction that might act to suppress fibrinolysis, complement activation and coagulation. Indeed, elevated levels of all three fibrinogen chains were detected in CIPF WHWTs. These proteins were consistent with an increase in plasma exudate in the BALF from CIPF patients, although the lack of a similar increase in the most abundant plasma proteins, such as albumin and transferrin, implied a degree of selectivity.

The proteins that were most significantly decreased in CIPF compared to healthy WHWTs included carboxylic acid ester hydrolase, non-enzymatic chitinase 3-like 1 and secretoglobulin. Carboxylic acid ester hydrolase is relevant to fatty acyl and sterol ester metabolism and capable of cleaving thioester or amide bonds. This enzyme is expressed in lung in secreted form but its precise role there is unknown [23]. Interestingly, the non-enzymatic chitinase 3-like 1 has been implicated in the inflammatory response to respiratory virus infection where it is elevated in the inflammatory phase [24], and in the fibroproliferative phase in mouse models of pulmonary fibrosis [25]. The decrease in this protein is thus somewhat of a contradiction. Secretoglobulin, secreted by club cells, is the most abundant protein in airway fluid and the decrease in CIPF is consistent with an impairment of lung function. Indeed, a decrease in secretoglobulin production could reduce the ability of lung to resist cytokine surges [26].

To establish whether WHWTs were aspirating gastrointestinal contents, we examined BALF for the presence of proteins that were putatively derived from the upper digestive tract. In our study, 126 proteins were identified in both gastric juice and induced vomit, which concurs with the median number of proteins in canine gastric fluid in a previous study [13]. In a previous study [6], we found bile acids, a commonly used marker of reflux aspiration, in BALF from both age matched CIPF and healthy WHWTs but not in healthy Beagles.

One commonly used protein marker of reflux aspiration in humans is pepsin [8]. We were able to detect peptides derived from pepsin in gastric juice and vomitus, even though pepsin is of relatively low abundance in canine gastric fluid [13] compared to human gastric fluid [27]. However, when we examined the liquid chromatography tandem mass spectrometry (LC-MS/MS) traces for the presence of these peptides in tryptic digests of BALF, they were no different to baseline signals, suggesting that these peptides were absent or below the lower limit of detection. The identification of pepsin by tryptic digestion and LC-MS/MS is difficult. It has disproportionately few basic amino acids as it is required to function in the acidic environment of the stomach lumen; it thus possesses a low isoelectric point [28]. Because trypsin cleaves proteins at lysine and arginine residues, most peptides generated from pepsin by tryptic digestion are incompatible with an LC-MS/MS workflow meaning that only around 7% of the pepsin sequence (two peptides) could be expected to be identified using this method.

We could find no strong evidence for the presence of other secreted gastric proteins in BALF. Trefoil factor family proteins, extracellular, secreted proteins widely expressed in mucin producing cells are abundantly expressed in the gastrointestinal epithelium [29]. These are robust proteins that are resistant to acid, and thus, the acidic milieu of the gastric contents. Three TFF proteins are reported in dog (TFF 1–3, [30]). TFFs have been proposed to play a role in mucosal defence and healing [29] and TFF1 is strongly expressed in canine stomach (https://bgee.org/?page=gene&gene_id=ENSCAFG00000010351). Although we were able to detect TFF1 in canine gastric juice and vomitus and in BALF samples of CIPF and healthy WHWTs, no significant differences were detected between groups. Similarly, gastrokines, stomach-specific, secreted proteins that interact with TFF proteins [31, 32]. could not be identified in BALF in healthy or CIPF WHWTs. One protein that was identified through multiple peptides, with both proteases in gastric juice and vomitus, was E2RH46_CANLF, a large, secreted, putative glycoprotein of ‘uncharacterised function’ that is flagged as being expressed in goblet cells. It might therefore meet the criterion of a suitable, stomach-derived protein to monitor reflux aspiration. This protein was detected in tryptic digests of BALF both in CIPF and healthy WHWTs but there was a broad distribution of values, and no difference in the abundance of this protein was observed between the two groups. Collectively, these data support the notion that reflux aspiration is challenging to detect in CIPF, as also noted by Grobman et al. [13]. However, group sizes were small. Also, only one gastric juice and one vomit sample were available and were not from WHWTs, although our data aligned well with an independent study [13]. Secondly, BALF samples from healthy dogs of other breeds than WHWT were not examined. Additionally, even though healthy WHWTs were thoroughly examined and considered to have healthy lungs, there is a possibility that some of them might have been in an early, subclinical stage of CIPF. Lastly, as with TFF1 and E2RH46 in this study, no significant difference in bile acid concentrations in BALF between CIPF and healthy WHWTs was detected in a previous study [6] leaving a possibility that also healthy WHWTs might aspirate refluxate. Therefore, adding two groups in comparison representing nonfibrotic lung disease and healthy dogs both of another breed might be informative.

In a conclusion, label-free proteomics allowed discrimination between CIPF and healthy WHWTs, consistent with inflammatory and fibrotic processes in lungs, but could not find clear evidence for reflux aspiration. Targeted measurement of key proteins identified herein may provide a proteomics signature of CIPF that could be used to evaluate treatment options and progress. However, evidence for reflux aspiration remains rather conjectural at this stage.

## Methods

**Fig. 6:**
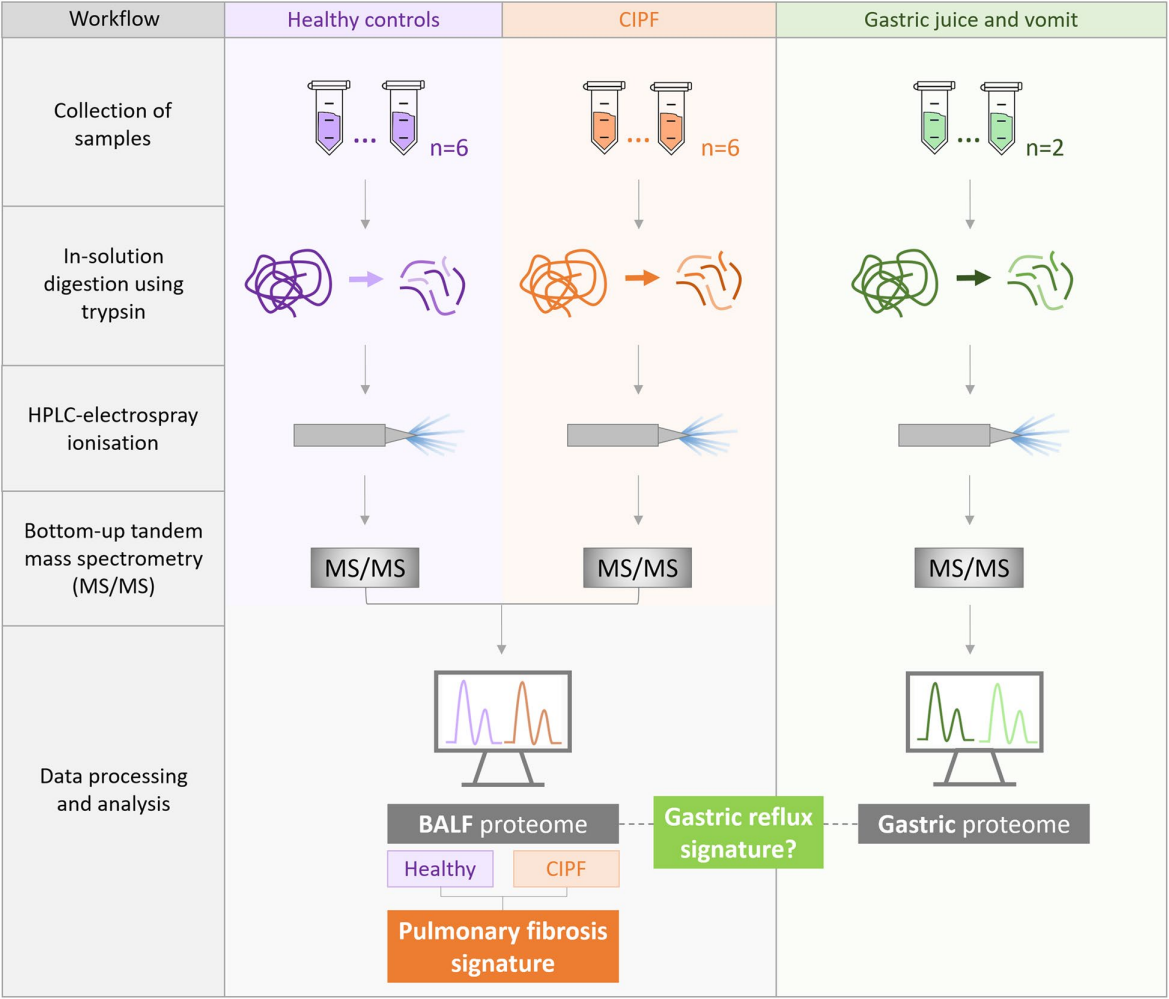
Sample processing and analytical workflow

The overall sample workflow is presented in Fig. 6. In the first part of this study, we evaluated proteomic profile of BALF samples from six CIPF and six healthy WHWTs. In the second part of this study, we conducted a proteome analysis of gastric juice and induced vomit from single individuals, with the goal of building a list of abundant, secreted gastrointestinal proteins that might be of value as markers of reflux aspiration. In this first pass, we allowed identifications that were based on single, high-quality peptide matches to maximise the list of candidates. Systematic exploration of the literature relating to these proteins and exploring the homologues in the Human Protein Atlas (https://www.proteinatlas.org/) revealed that the majority of these would not provide the tissue specificity to allow them to be used to assess reflux aspiration. Putative candidates were then used to focus a search in BALF samples in CIPF and healthy WHWTs, (*n* = 6, both groups).

### Study subjects

A total of 6 CIPF WHWTs (median age 11.6 years) and 6 healthy WHWTs (median age 8.3 years were retrospectively selected for this study (Supplementary Table 4). All WHWTs were thoroughly examined; clinical workup included physical examination, hematology, serum biochemistry, arterial blood gas analysis, fecal sample analysis, cervicothoracic radiographs, bronchoscopy with BALF sampling and high-resolution computed tomography. CIPF diagnosis was verified with postmortem lung histopathology in all dogs. Owners reported no signs indicating gastrointestinal disease in any of the dogs at the time of sample collection. Additionally, two healthy dogs of other breeds were included in this study (gastric juice sample: Belgian shepherd, 7.5 years, female and vomit sample: Australian shepherd, 10.9 years, female). All dogs were client-owned pet dogs and were examined in the Veterinary Teaching Hospital of Helsinki University.

### Collection of samples

Bronchoscopy and BALF sampling were performed under general anesthesia. BALF samples were collected from left and right caudal lung lobes using sterile saline (2 ml/kg/lobe). Supernatant was separated by centrifugation and stored at -80 °C [33]. Gastric fluid sample was collected during gastroscopy by flushing with saline and vomit sample by inducing vomiting with apomorphine (0.2 mg/kg). Both were stored similarly as BALF samples.

### Rapigest in-solution digestion

BALF, gastric juice and vomit samples were diluted in 25 mm ammonium bicarbonate to a final volume of 160 µl to yield a total protein concentration between 0.11 and 0.13 mg/ml. Samples were treated with a 1% (w/v) solution of Rapigest SF Surfactant (Waters) to a final concentration of 0.05% (w/v) before incubation at 80 °C for 10 min. Disulfide bonds were reduced by the addition of dithiothreitol (DTT) to a final concentration of 3 mM and incubated for 10 min at 60 °C. Iodoacetamide (IA) was added to a final concentration of 9 mm to alkylate the free sulfhydryl groups on the cysteine residues. After incubation for 30 min at room temperature in the dark, trypsin was added to a 50:1 protein: protease ratio and incubated overnight at 37 °C. Digestion was stopped by the addition of trifluoroacetic acid (TFA) to a final concentration of 0.5% (v/v) and incubated for 45 min at 37 °C. The samples were centrifuged to remove particulates.

### Liquid chromatography-mass spectrometry

Peptides were analysed by LC-MS/MS using an Ultimate 3000 nano system (Dionex/Thermo Fisher Scientific, Hemel Hempstead, UK) coupled to a Q-Exactive-HF mass spectrometer (Thermo Fisher Scientific, Hemel Hempstead, UK) to acquire the masses of peptides derived from proteins extracted from the BALF samples. Peptides were loaded onto a trap column (50 cm) (Acclaim Pepmap 100) at 12 µl/min over 7 min with an aqueous solution containing 0.1% (v/34v) TFA and 2% (v/v) acetonitrile. The trap column was set in-line with an analytical column (Easy-Spray Pepmap® RSLC) (Dionex). Peptides were eluted by applying a linear gradient of 3.8–50% solvent B (acetonitrile 80% (v/v) with 0.1% (v/v) formic acid FA over 35 min at 300 nl/min. Solvent A was HPLC grade water with 0.1% (v/v) formic acid. The mass spectrometer was operated in data dependent positive (ESI+) mode and full scan MS spectra (350–2000 m/z) were acquired in the Orbitrap. The 16 most intense multiply charged ions (z ≥ 2) were sequentially isolated and fragmented in the octopole collision cell by high energy collisional dissociation (HCD) and detected in the Orbitrap.

### Protein identification and analysis

Samples were searched against a database comprised of all canine protein sequences in the Uniprot Uni_Canine database (45,397 proteins) using trypsin as the specified enzyme, carbamidomethylation of cysteine as fixed modification, methionine oxidation as variable modification and one trypsin missed cleavage, precursor and fragment ion error tolerances were set to 10 ppm and 0.01 Da, respectively. The false discovery rate (FDR) was calculated using the decoy database tool in MASCOT. Only those proteins identified with an FDR < 1% were accepted. Label-free quantification, including run alignment and peak picking, was carried out in Progenesis QI for Proteomics v4 (Nonlinear Dynamics), while database searching was carried out by MASCOT search engine (Matrix Science version 2.6.0.). The data exported from Progenesis was imported into Simplifi (simplifi.protofi.com) for rapid visualisation and calculation of non-parametric statistics and the output table from Simplifi is presented as Supplementary Table 3. The Simplifi visualisation of these data is available at https://simplifi.protifi.com/#/p/f1d8cd90-2416-11eb-8cd5-fb853c2621cb. Functional enrichment analysis of BALF core proteome were performed using g:Profiler (https://biit.cs.ut.ee/gprofiler/gost). The mass spectrometry proteomics data have been deposited to the ProteomeXchange Consortium (http://proteomecentral.proteomexchange.org) via the PRIDE partner repository [34] with the dataset identifier PXD028916.

### Statistical analysis

Data were analysed and visualised in the R environment, using RStudio version 4.0.3 (updated 2020-10-10). Heatmap with hierarchical clustering and principal component analysis (PCA) was completed in RStudio using heatmap3 and prcomp functions, respectively. Protein abundance data were log transformed for both analyses. The PCA data was additionally univariance scaled.

## Supplementary Information

Additional file 1: **Supplementary Figure 1.** Abundance profiles for the 20 proteins making the largest contributions to PC2 in the principal components analysis (Figure 4, main text).

Additional file 2: **Supplementary Figure 2.** Abundance profiles for the 40 proteins with the largest fold differences (Figure 4b, main text).

Additional file 3: **Supplementary Table 1.** The core BALF proteome.

Additional file 4: **Supplementary Table 2.** Gastric juice and induced vomit proteins.

Additional file 5: **Supplementary Table 3.** Output table from Simplifi.

Additional file 6: **Supplementary Table 4.** Patient data.

## Data Availability

The mass spectrometry proteomics data have been deposited to the ProteomeXchange Consortium (http://proteomecentral.proteomexchange.org) via the PRIDE partner repository [34] with the dataset identifier PXD028916. Username: reviewer_pxd028916@ebi.ac.uk. Password: fGhtVAcA

## Abbreviations

BALF: Bronchoalveolar lavage fluid
CIPF: canine idiopathic pulmonary fibrosis
IPF: idiopathic pulmonary fibrosis
LC-MS/MS: liquid chromatography tandem mass spectrometry
WHWT: West Highland white terrier
