# Supplementary figures and images for: Quantitative proteomic analysis of bronchoalveolar lavage fluid in West Highland white terriers with canine idiopathic pulmonary fibrosis
Source: BMC Vet Res. 2022 Mar 30;18:121. doi: 10.1186/s12917-022-03202-x (PMC8966175; doi:10.1186/s12917-022-03202-x)

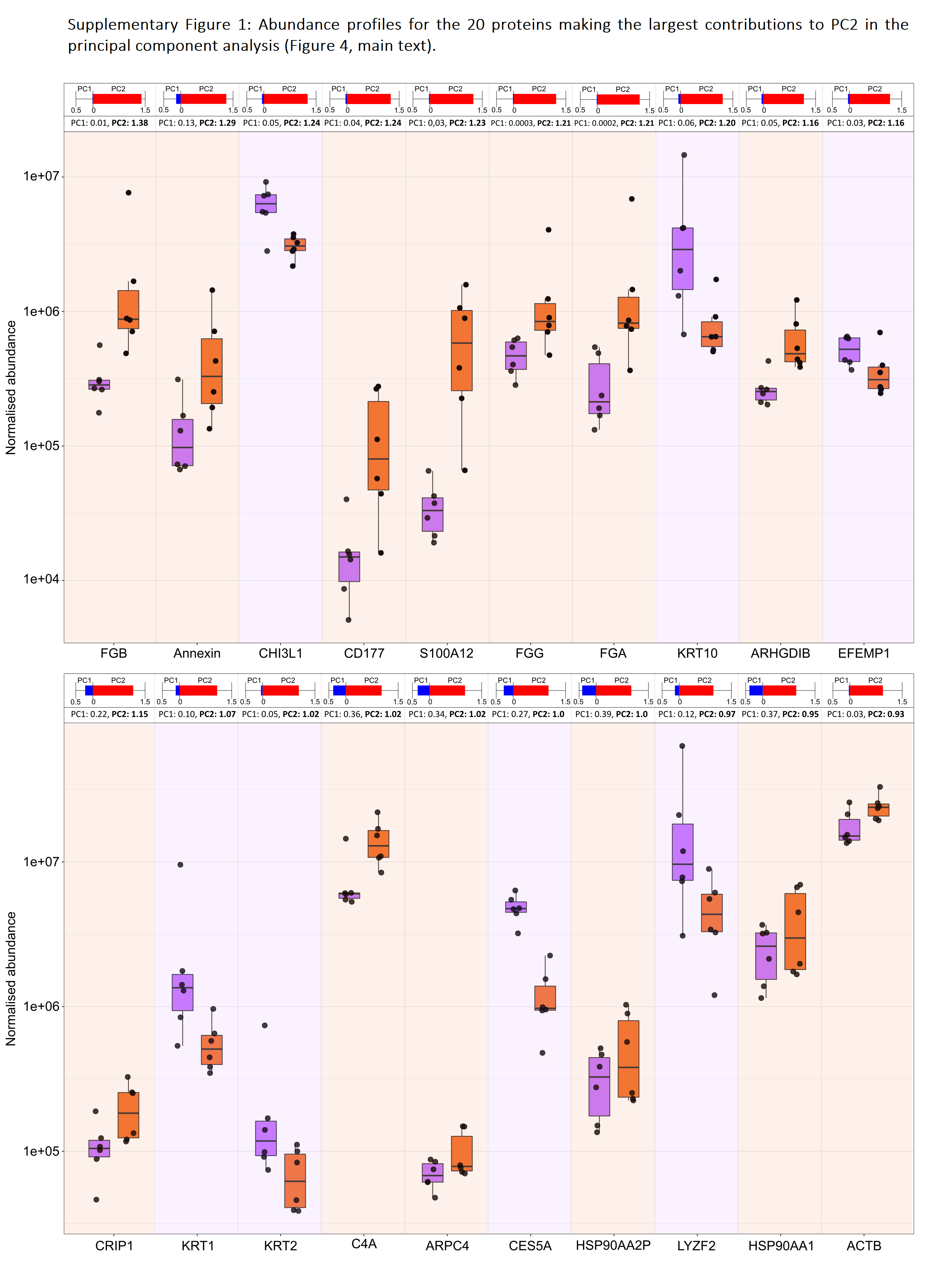

Supplement: Supplementary file 1 — Supplementary Figure 1. Abundance profiles for the 20 proteins making the largest contributions to PC2 in the principal components analysis (Figure 4, main text). [file 12917_2022_3202_MOESM1_ESM.png]

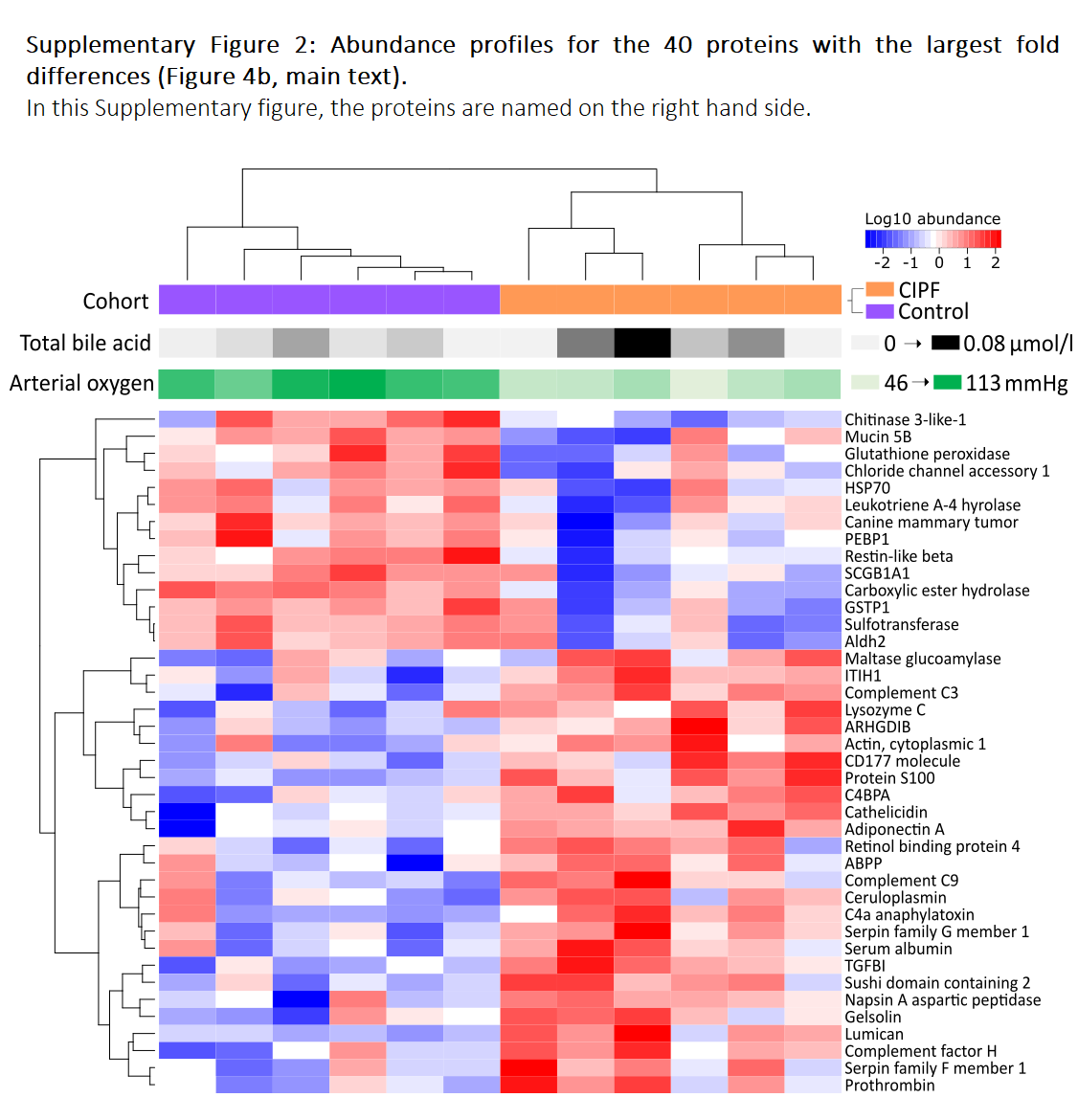

Supplement: Supplementary file 2 — Supplementary Figure 2. Abundance profiles for the 40 proteins with the largest fold differences (Figure 4b, main text). [file 12917_2022_3202_MOESM2_ESM.png]
